# A Few Grains and Some Chaff, from an Old Country Doctor

**Published:** 1886-07-20

**Authors:** 


### A FEW GRAINS AND SOME CHAFF.

### FROM AN OLD COUNTRY DOCTOR.

*Rheumatism.*—Phodophyllin, given in small and repeated doses, I have found very beneficial in rheumatism. This remedy was first recommended by some one about the time that salicylic acid began to attract attention ; and the latter so eclipsed the former that but little attention was paid to it. It is, however, a good remedy, judging from my limited experience. Podophyllin, grains iv; ipecac, grains iij ; camphor, pulverized, grains xx, make 12 pills ; give one pill every hour until active purgation is induced. Use Dover's powder to relieve pain. This, however, is best omitted until after the purgative effect of the pills ; but if the pain requires it, give previously. I was astonished at the prompt relief afforded by this treatment.

*Scabies.*—During the war, one K—— returned home with the above disease. He was told that it was camp lichen—that it was not contagious, and would get well of itself before he had been at home a week. The young and pretty wife of K—— (he had been married a year) began to complain, and he made his troubles known to one Early G——, the then reputed oldest inhabitant of this section. Early put on his spectacles and, after a careful examination, told K—— that he had the "*each*" sure, and the seven year "each" at that. "Well," said K——, "what must I do for it?" "O," said Early, "it is easily cured?" "How?" "To-night at bedtime," said Early, "make a good fire in your room, but between now and then mix you a good chance of sulphur and grease, and strip naked, you and your wife, before the fire, and you anint your wife; and let your wife anint you. Do this for a week, and don't wash until the end of the week, and I'll be dry shaved with a spoonhandle if it don't cure you." K—— tried Early's prescription faithfully, but it failed. I, like K——, happened to be at home on leave of absence, so he applied to me, and told me of the above prescription and directions of Early. I made him the following prescription: ℞. Toilet soap, cut into thin shavings, half a cake; boiling water sufficient to make, by rubbing in a mortar, a thin paste; add sulphur, rubbed fine previously in a mortar, sufficient to make the paste quite stiff. I prepared this myself and directed the patient to wash the entire surface, the unaffected as well as the affected parts, with *hot* water and soap, and then to apply the liniment to the whole surface, using hot water enough to make the liniment apply easily. Repeat this every night for a week, at the end of which time K—— reported both cases cured.

I had almost forgotten the above incident, when, about a week ago, an entire family afflicted with the same annoying disease came under my care. The same prescription promptly cured them all. The liniment is much more agreeable than the sulphur ointment, and of equal efficacy.

*Neuralgia, Note 1.*—℞. Ferri sulph., grs. iv; quinia sulph., grs. vi; extract belladonna, grs. v. M. Fiat pill No. xii. Give one pill three times a day. If the patient is constipated, a cathartic should precede the above, or, if habitually of costive habit, I add to the prescription. podophyllin, grs. iij, and if hemorrhoids do not afflict the patient, aloes soct., grs. xij; if they do, substitute extract jalap, same quantity. These pills will be too small without the aloes or jalap, and conserve of roses should be added to make them of proper size.

The following incident occured to the writer several years ago, after making this precription: A patient fresh from the wiregrass region applied for relief for "neuralgia." I, after administering a purge, made him twenty four of the above pills. He paid me $1.50, saying it was "a pretty steep charge for a box of pills and a dose of salts," and left. He returned in a few days, saying he was "all right—cured on half the pills," the other half he insisted on my taking back and returning him seventy-five cents! What is a doctor to do under such fire as that? My first impulse was to kick the ass out of my office; but remembering that asses were themselves given to kicking, decided to keep cool. I found it was a waste of time to try to make the numskull understand that my charge was for the relief afforded him, and not so much for the pills, but this seemed entirely too fine a point for his comprehension. Our city brethren, who have trained their patrons to get prescriptions at the drug store, know but little of the trouble and annoyance of village and country doctors on this point and many others.

*That Bile.*—A man hobbled into my office one morning, and after seating himself, said: "Doctor, I have a *bile* on my leg that pains me very much; please examine it, and tell me what to do for it." On examination, I found a small furuncle just below the knee; "O," said I, "apply some slippery elm bark to it and it will soon be ready to open." He left, and the next day returned, hobbling still worse, saying: "Doctor, I don't think I can possibly stand the slippery elm treatment any longer; I have got worse ever since I applied it." I asked him to let me see it. On examination, I found he had applied a piece of elm bark, about an inch square, firmly to the part with a bandage. I made a poultice of elm bark and applied it properly, telling him that was what I meant the day before. "Well," said he, "I generally try to follow instructions just as I get them, and I don't want no palaver and big words in mine." I relate this circumstance merely to impress upon the minds of my junior brethren the importance of being very plain and full in giving instructions to ignorant patients.

*Burns.*—I have long used charcoal in burns, and am convinced that it is the best external application I ever used. In treating these painful injuries, I exclude as far as possible oxygen, the heat-sustaining element, and persistently apply carbon, its opposite. I have, by this treatment, cured cases that our best authorities pronounced incurable, and which were so, perhaps, under any other treatment. I proceed thus: Take of albumen of egg quantity sufficient to make a thin poultice, with powdered charcoal, large enough to cover the affected part, and to extend a little distance beyond it. Having applied this, I prepare a similar poultice and, in fifteen minutes, remove the first and apply the second. A bit of any thin, loosely-woven fabric should cover the applied side or face of the poultice. On removing the poultice, remove this, and mix the poultice, adding a little more of the albumen if necessary. Continue this, changing the poultices. The relief afforded by this application is simply astonishing. The only objection to the application is that, if the cuticle is removed, a stain occasionally remains after the part is healed; for this reason, the cuticle should be punctured and the serum removed, and the cuticle otherwise as little disturbed as possible. Soda owes its virtues to the carbon it contains. The albumen makes a more consistent poultice, and is the best application that can be used; water is not suitable.

This poultice is also the best local application I am acquainted with for erysipelas. If the face, neck or arms is the part injured, for fear of the stain, some other application, as soda, had best be used ; but the prompt relief afforded by the first application places it far ahead of all others.

